# Mathematical modeling of bone remodeling after surgical menopause

**DOI:** 10.3389/fsysb.2026.1729027

**Published:** 2026-02-11

**Authors:** Anna C. Nelson, Edwina F. Yeo, Yun Zhang, Carley V. Cook, Sophie Fischer-Holzhausen, Lauryn Keeler Bruce, Pritha Dutta, Samaneh Gholami, Brenda J. Smith, Ashlee N. Ford Versypt

**Affiliations:** 1 Department of Mathematics and Statistics, University of New Mexico, Albuquerque, NM, United States; 2 Department of Mathematics, University College London, London, United Kingdom; 3 Biomechanics Section, Department of Mechanical Engineering, KU Leuven, Leuven, Belgium; 4 School of Biological Science and Medical Engineering, Southeast University, Nanjing, China; 5 Department of Chemical and Biological Engineering, University at Buffalo, The State University of New York, Buffalo, NY, United States; 6 ESQlabs GmbH, Saterland, Germany; 7 UC San Diego Health Department of Biomedical Informatics, University of California San Diego, San Diego, CA, United States; 8 Department of Applied Mathematics, University of Waterloo, Waterloo, ON, Canada; 9 Modelling Infection and Immunity Lab, Centre for Disease Modelling, Mathematics and Statistics, York University, Toronto, ON, Canada; 10 Indiana Center for Musculoskeletal Health, Indiana University School of Medicine, Indianapolis, IN, United States; 11 Department of Obstetrics and Gynecology, Indiana University School of Medicine, Indianapolis, IN, United States; 12 Department of Biomedical Engineering, University at Buffalo, The State University of New York, Buffalo, NY, United States; 13 Department of Pharmaceutical Sciences, University at Buffalo, The State University of New York, Buffalo, NY, United States; 14 Institute for Artificial Intelligence and Data Science, University at Buffalo, The State University of New York, Buffalo, NY, United States

**Keywords:** bone mineral density, bone remodeling, mathematical modeling, osteoporosis, surgical menopause, systems biology

## Abstract

Osteoporosis is a skeletal pathology characterized by decreased bone mass and structural deterioration resulting from an imbalance in bone metabolic processes. Estrogen deficiency in postmenopausal women leads to an increased risk of osteoporosis, while women who have undergone complete oophorectomies display an even higher risk due to the sudden decrease in estrogen. Some evidence indicates that bone loss slows in the period beyond 15 years after surgery; however, there is substantial uncertainty in clinical data. To explore the effects of surgically induced menopausal transition, here we propose a mathematical model for the bone cell dynamical responses to sudden estrogen deficiency, which extends an existing model for osteoporosis due to aging and natural menopause. Using data on key effects observed in female mice and humans after bilateral oophorectomy, this new model considers the role of osteocytes embedded within the mineralized bone matrix in regulating bone remodeling, which results in net bone loss after surgical menopause. The model parameter values in natural and surgical menopause were estimated from aggregated human clinical data from existing longitudinal studies. The new model effectively captures the previously unmodeled increase in bone loss during the first 15 years post-surgical menopause and the rebound in bone mineral density in the long-term. With this model, effects of treatments on targeting osteocyte dynamics could be explored in the future.

## 1 Introduction

Bone tissue is continuously resorbed and formed through the bone remodeling process, which maintains healthy tissue and repairs micro-fractures in the skeleton. At homeostasis, the biomechanical, biochemical, and cellular mechanisms involved in remodeling of the adult skeleton maintain bone mass. However, any alteration in this complex bone turnover cycle can result in changes in bone (i.e., pathologic or anabolic) (Allen and Burr, 2014). The cellular mechanisms that determine bone health occur in functional locations called basic multicellular units (BMUs) within which cellular interactions contribute to bone tissue remodeling through a continuous cycle of activation, resorption, and formation (Robling et al., 2006). The three main types of cells that contribute to this cycle are osteoclasts, osteoblasts, and osteocytes. Osteocytes, widely recognized for their strain sensitivity, are the most abundant of these cells and signal to recruit other cells to the BMU to initiate bone resorption and formation (Creecy et al., 2021). One such signal is sclerostin (Suen and Qin, 2016; Plotkin and Bellido, 2016; Delgado-Calle et al., 2017), which negatively regulates bone formation by inhibiting Wnt (Li et al., 2005; Krause et al., 2010; Atkins et al., 2011; Bellido, 2014; Kim et al., 2020), thus reducing osteoblastogenesis. Sclerostin has also been shown to contribute to bone resorption by upregulating secretion of receptor activator of nuclear factor κ B (RANK) ligand (RANKL) from osteocytes (Wijenayaka et al., 2011; Suen and Qin, 2016), which stimulates osteoclastogenesis (Nakashima et al., 2011; Tomkinson et al., 1998; Fujiwara et al., 2016; Plotkin and Bellido, 2016; Karlafti et al., 2019). Osteoclasts degrade the bone protein matrix and solubilize the mineral hydroxyapatite, and osteoblasts initiate bone matrix formation by forming osteoid tissue, which is later mineralized into bone (Kenkre and Bassett, 2018). Up to 20% of the osteoblasts within osteoid tissue differentiate further into osteocytes (Parfitt, 1976; Martin et al., 1998; Plotkin and Bellido, 2016; Delgado-Calle and Bellido, 2022). After the resorbed bone tissue is replaced through bone formation, osteocytes signal to BMU cells to slow bone formation (Kenkre and Bassett, 2018).

Perturbations of the bone remodeling process can cause an imbalance between catabolic and anabolic activity, leading to bone pathologies characterized by substantial bone loss. In particular, osteoporosis is a low-bone-density disease caused by such an imbalance and leads to increased fracture risk, reducing quality of life for those affected and imposing a significant financial burden on the global economy (Harvey et al., 2010). Osteoporosis is prevalent in postmenopausal women, with estimates suggesting that between 30% and 40% of women over age 50 are affected by low bone mass or osteoporosis (Rachner et al., 2011; Sarafrazi et al., 2021). Estrogen-deficient bone loss has been linked to several metabolic processes in bone remodeling. The presence of estrogen has been shown in human studies to prevent apoptosis of osteocytes and osteoblasts, and estrogen reduces levels of sclerostin (Mödder et al., 2010). Estrogen also reduces the impact of osteoclasts by preventing osteoclastogenesis and increasing osteoclast apoptosis (Florencio-Silva et al., 2015). When estrogen levels temporarily drop during perimenopause or are permanently low after menopause, osteoclast differentiation increases and causes more bone removal (Hsu et al., 2024); meanwhile, despite an initial increase in osteoblasts with the decline in estrogen, the bone-forming osteoblast activity is unable to match the pace of the increase in resorption, resulting in less bone mass (Jilka et al., 1998; Almeida et al., 2007; Khosla, 2013; Seeman, 2013; Karlamangla et al., 2021).

For women with intact ovaries, highly variable and declining estrogen production throughout perimenopause and menopause leads to increased bone loss. During late perimenopause and the early postmenopausal period, human cohort studies have shown that bone is lost at a rate of 2%–2.4% per year in the spine and 1.2%–1.7% per year in the hip (Finkelstein et al., 2008; Shieh et al., 2016). During this time, women lose approximately 25% of their trabecular bone (honeycomb-shaped bone structures) and 15% of their cortical bone (dense bone tissue on the outside of bone structures) (Finkelstein et al., 2008). This rapid bone degradation lasts about 5–10 years. After this period, bone is lost at a much slower rate of 0.5% per year (The North American Menopause Society, 2021).

Another cause of estrogen loss is the surgical removal of the ovaries. A bilateral oophorectomy is usually performed to reduce the risk of cancer or treat non-malignant ovarian diseases, such as endometriosis or benign cysts (Cohen et al., 2012; Challberg et al., 2011; Aitken et al., 1973). This sudden onset of estrogen deficiency increases a patient’s risk for osteoporosis (Rodriguez and Shoupe, 2015). After oophorectomy, both the age of the patient at surgery and the usage of hormone replacement therapies influence the likelihood of developing osteoporosis. Oophorectomy before age 45 has been reported to increase the risk of osteoporosis; however, those who underwent an oophorectomy after age 45 had bone density similar to that of women with intact ovaries (Aitken et al., 1973). A review of available early postmenopause data found that the earlier menopause occurred, whether natural or surgical, the lower the resulting bone density became (Gallagher, 2007). Abnormal bone scans were identified in 71% of women who underwent a preventative bilateral oophorectomy (Cohen et al., 2012). This study did not find a difference between those who underwent surgery before or after the age of natural menopause; however, the authors pointed out that there was a large difference in follow-up ages between the two groups, indicating that the age of the woman when the bone scans were taken is also an important factor that should have been accounted for (Cohen et al., 2012). Fakkert et al. (2017a) provided a systematic review of bone mineral density (BMD) following surgical menopause. They cautioned about data bias in the reported data. They concluded that while surgical menopause substantially decreases BMD, this decline becomes indistinguishable from that observed after natural menopause, once the age of natural menopause is reached (Fakkert et al., 2017a). Oophorectomy-induced bone loss may be prevented with hormone replacement therapy, but many women have an aversion to taking estrogen due to perceived risk (Challberg et al., 2011). Overall, the etiology of bone loss leading to osteoporosis due to surgical menopause needs further exploration.

The impacts of surgical menopause on mechanisms involved in bone remodeling have been explored using ovariectomized animal models, where animals undergo either an ovariectomy (OVX) procedure or a control procedure (i.e., sham operation) that mimics surgery but keeps the ovaries intact, and via *in vivo* studies from human biopsies or animal cells in culture. Several studies have shown that osteocyte apoptosis increased after estrogen withdrawal using *in vitro* experiments (Brennan et al., 2014); sheep, rat, and mouse *in vivo* experiments (Brennan et al., 2011; Tomkinson et al., 1998; Huber et al., 2007; Emerton et al., 2010; Florencio-Silva et al., 2018); and *ex vivo* experiments in human bone (Tomkinson et al., 1997). In *in vivo* animal studies, this increased apoptosis persisted for at least 31 months post-OVX (Brennan et al., 2011). However, the study by Florencio-Silva et al. (2018) found differing results: the number of osteocytes was significantly lower only immediately after surgery, and osteocyte counts increased by 1 month post-OVX. Osteocyte death has been proposed as a key marker of poor bone quality (Milovanovic and Busse, 2020), and the OVX experiments mentioned above suggest a marked change in osteocyte number after surgical menopause in contrast to natural menopause. Other *in vitro* animal studies showed that estrogen-deficient osteocytes release higher levels of RANKL (Sipos et al., 2009; Hofbauer et al., 2004), which stimulates the differentiation rate of osteoclast precursors (McNamara, 2021; Naqvi et al., 2020; Choi et al., 2008). It has not yet been quantified how sudden estrogen loss interacts with all cell types in combination to affect overall BMD and fracture risk.

Although estrogen plays a central role in bone health, few mathematical models in systems biology focus on the mechanisms of estrogen’s impact on bone (Cook et al., 2024). There is extensive literature on mathematical models of bone remodeling, as reviewed by Pivonka and Komarova (2010) and Cook et al. (2024). Most models of biochemical and cellular species dynamics use either power-law approximations (Komarova et al., 2003; Graham et al., 2013; Cook et al., 2022) or mass-action kinetics (Lemaire et al., 2004; Pivonka et al., 2008). Other models focus on how mechanical loading and morphology can affect bone remodeling (Lerebours et al., 2016; van Oers et al., 2008; Scheiner et al., 2013; 2014; Larcher and Scheiner, 2021). A recent review from our team (Cook et al., 2024) identified published mathematical models that incorporate explicit effects of estrogen in bone remodeling and postmenopausal treatments (Rattanakul et al., 2003; Schmidt et al., 2011; Post et al., 2013; Berkhout et al., 2015; 2016; Chaiya and Rattanakul, 2017; Javed et al., 2018; Jörg et al., 2022) and others with implicit estrogen effects (Lemaire et al., 2004; Scheiner et al., 2013; 2014; Lemaire and Cox, 2019; Trichilo et al., 2019; Martin et al., 2019; Larcher and Scheiner, 2021). In particular, these models investigate postmenopausal osteoporosis by adjusting parameters affected by estrogen decline or senescence. Many of these parameters describe mechanisms in the RANKL pathway (Pivonka et al., 2010; Lemaire et al., 2004; Scheiner et al., 2014; Lemaire and Cox, 2019; Martínez-Reina et al., 2021). A recent paper by Ruiz-Lozano et al. (2024), published after our review, distinguishes the effects of aging from those of estrogen decline by examining men and women. For aging, the model includes the effects of increased production of sclerostin and its impact on Wnt signaling and anabolic and catabolic effects of transforming growth factor (TGF)- β , and estrogen loss is modeled implicitly via time-dependent RANKL and osteoprotegerin (OPG) changes. Our review (Cook et al., 2024) provides further details on the cells and signaling molecules included in various models, and an overview of the network of complex signaling interactions involving sclerostin, Wnt, and RANK-RANKL-OPG, along with their cellular sources. Beyond estrogen effects, models that incorporate osteocyte effects (Graham et al., 2013; Martínez-Reina et al., 2021; Cook et al., 2022; Jörg et al., 2022; Ruiz-Lozano et al., 2024) show promise in capturing age- or menopause-related changes in osteocyte signaling.

While mathematical modeling has been useful for studying postmenopausal osteoporosis, none of these models explicitly considers the effects of surgical menopause on bone cell populations. The recent model by Jörg et al. (2022) includes estrogen’s effects on osteoclasts and sclerostin, includes resorption signals (lumped effects for TGF- β , bone morphogenetic protein, and the RANK-RANKL-OPG pathway, among others), and incorporates osteocyte dynamics. This model is based on realistic human time frames and includes pharmacological treatments. In particular, the mathematical model is parameterized using BMD data from patients who experienced natural menopause (Looker et al., 1998) and estimates parameters using datasets that incorporate different treatment protocols. However, the natural menopause data used to parameterize this model contains only two postmenopausal data points. Furthermore, as in the other models discussed in Cook et al. (2024), the model in Jörg et al. (2022) does not consider an abrupt decline in estrogen, which occurs in the surgical menopause scenario, nor does this model incorporate important mechanisms involved in surgical menopause, such as the impact of osteocyte death on BMD. A few prior models allow for osteocyte death (Graham et al., 2013; Cook et al., 2022), but they do not account for the effects of estrogen or aging. A limitation of most previous models is that they do not account for osteocyte apoptosis (Martínez-Reina et al., 2021; Ruiz-Lozano et al., 2024) or for osteocyte changes at all. While osteocytes are long-lived, osteocyte apoptosis is upregulated in estrogen deficiency (Tomkinson et al., 1997; 1998; Milovanovic and Busse, 2020). So, incorporating this mechanism is important for activating bone remodeling (Khosla et al., 2012), which is enhanced in surgical menopause (Peris et al., 1999; Fakkert et al., 2017b; Matsuno et al., 2025).

In this paper, we (1) aggregate BMD data sources from natural menopause patients and then reparameterize a subset of parameters in the Jörg et al. (2022) model to ensure that the mechanisms of the model reflect the broader BMD trends after natural menopause, (2) include new estrogen dynamics to describe the sudden and dramatic loss of estrogen due to surgical menopause, and (3) extend the mathematical Jörg et al. (2022) model for the dynamical responses of BMU bone cells to the case of estrogen deficiency during the surgical menopausal transition using information about the critical impacts observed in female mice and humans after removal of the ovaries. The new model considers the role of embedded osteocytes in regulating osteoclast differentiation and inducing enhanced bone resorption after surgical menopause. With this new model, we perform parameter exploration to determine which mechanisms are most important for capturing trends in surgical menopause data. This model could be used to explore medical interventions to correct the imbalances in bone remodeling after surgical menopause in a population at higher risk for early onset of osteoporosis.

## 2 Methods

### 2.1 Curated bone mineral density (BMD) data

While the impact of gradual estrogen decline on the bone remodeling process was investigated by Jörg et al. (2022), the dataset used to parameterize their model was sparse after menopause (Looker et al., 1998). In the Looker et al. (1998) dataset, only two BMD measurements from the proximal femur were recorded after menopause; furthermore, these data were aggregated by age group and did not specify menopause onset, which can lead to underestimates in BMD loss. To improve the accuracy of the Jörg et al. (2022) model in the natural menopause scenario and to parameterize our new surgical menopause model, we gather a larger set of published postmenopausal data. Due to the available datasets, we aggregate lumbar spine measurements from women without hormonal replacement for both natural and surgical menopause. In Table 1, we show data aggregated from cross-sectional studies that measured BMD using dual-energy X-ray absorptiometry in women undergoing both surgical and natural menopause.

**TABLE 1:** Summary of bone mineral density (BMD) data curated for parameterization of model. Data are taken from cross-sectional studies that use dual-energy X-ray absorptiometry to measure BMD.

Study source	BMD source	Number of women (type of menopause)
Pansini et al. (1995)	L2-L4 vertebrae	160 (natural), 67 (surgical)
Ohta et al. (2002)	L2-L4 vertebrae	20 (natural), 20 (surgical)
Hibler et al. (2016)	L1-L4 vertebrae	53 (surgical)
Hadjidakis et al. (2003)	L2-L4 vertebrae	177 (natural), 210 (surgical)
Chittacharoen et al. (1999)	L1-L5 vertebrae	309 (natural), 102 (surgical)
Chittacharoen et al. (1999)	L2-L4 vertebrae	141 (surgical)

**FIGURE 1:**
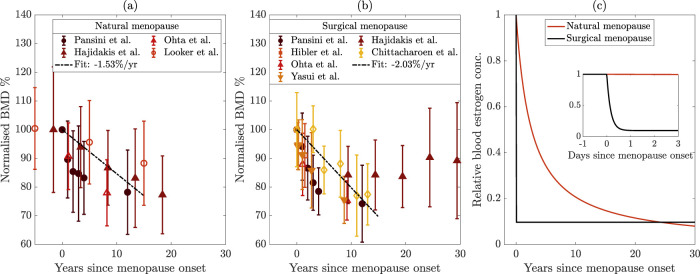
**(a,b)** Bone mineral density (BMD) measured in the lumbar spine of women (except Looker et al. (1998) are from the hip). Data are normalized to premenopausal levels from the time of menopause onset, and error bars represent the standard deviation. Normalization process for each dataset is detailed in Section 2.1. The data are from Pansini et al. (1995); Ohta et al. (2002); Hadjidakis et al. (2003); Looker et al. (1998); Hibler et al. (2016); Chittacharoen et al. (1999); Yasui et al. (2007). Dashed lines show linear fits to the data with slopes listed in the legends. **(a)** Natural menopause and **(b)** surgical menopause. **(c)** Comparison of estrogen decline in natural and surgical menopause, shown over 30 years after menopause onset. The inset shows the decline over the 3 days following menopause onset.

To compare BMD measurements across datasets, each dataset’s BMD values are normalized by their respective values at menopause onset, and time is rescaled to the age at menopause onset, defined as $t_{m}$ . The rescaled time is $t-t_{m}$ , where t is age in years. We plotted the average normalized BMD measurements and standard deviations for natural menopause (Figure 1a) and surgical menopause (Figure 1b). Motivated by linear bone loss estimates from cohort studies, we provide illustrative linear fits to data from the first 15 years postmenopause, but note that these fits were not used in our model. The slopes show a 1.54% decrease in BMD per year in the first 15 years for natural menopause (Figure 1a) and a 2.03% reduction in BMD per year over the same period for surgical menopause (Figure 1b). We note that the rate BMD loss postmenopause varies between different locations in the body with the lumbar spine having a reportedly greater loss than the femur per year (initially 1.67% and 3.12% loss per year, respectively) (Zhai et al., 2008); this is reflected in the difference between our aggregated datasets and Looker et al. (1998) (Figure 1a). Interestingly, the BMD measurements from Hadjidakis et al. (2003) suggest a rebound in BMD long after surgical menopause. We lack lumbar spine data from natural menopause due to the women’s advanced ages. The longest observational study measured femoral bone BMD up to 25 years postmenopause (Moilanen et al., 2020). The authors found that at this location in the body, bone loss during natural menopause occurs at a steady rate, with a total loss of 10% relative to baseline. Because we lack lumbar spine data for 20 years after natural menopause, it is unclear whether BMD slows or rebounds only in the lumbar spine after surgical menopause, or whether it also occurs after natural menopause.

### 2.2 Modeling bone remodeling after natural and surgical menopause

To understand how natural and surgical menopause differentially impact the bone remodeling system, we build on the mathematical model in Jörg et al. (2022), which assumes well-mixed chemical and cellular species within a BMU. Under this assumption, the dynamics of bone cell populations, chemical signals, and hormones are described by ordinary differential equations (ODEs). These species ultimately affect bone formation by altering the rates of bone production and degradation. First, we describe the model in Jörg et al. (2022), which tracks the cell densities of preosteoblasts, osteoblasts, preosteoclasts, osteoclasts, and osteocytes, as well as the sclerostin concentration, total bone density, and the bone mineral content (BMC). The precursor cells are continuously replenished and undergo apoptosis, which is influenced by various chemical signals. Osteoblasts may differentiate into osteocytes, which produce a chemical signal, sclerostin. Sclerostin upregulates osteoclast differentiation and downregulates osteoblast and osteocyte differentiation, thereby affecting bone density. The roles of the signalling molecules Wnt and RANKL are not explicitly included in the model; instead, their effects are captured by the impacts of sclerostin, and other signaling models were lumped into the resorption signals. Several estrogen effects were included in the Jörg et al. (2022) model: the inhibition of sclerostin production (Mödder et al., 2010) and the downregulation of osteoclastic bone resorption via suppressing osteoclast differentiation (Kameda et al., 1997). Therefore, a decrease in estrogen, through natural or surgical menopause, increases osteoclast and sclerostin levels and reduces osteoblast levels, which collectively lead to bone loss.

**FIGURE 2:**
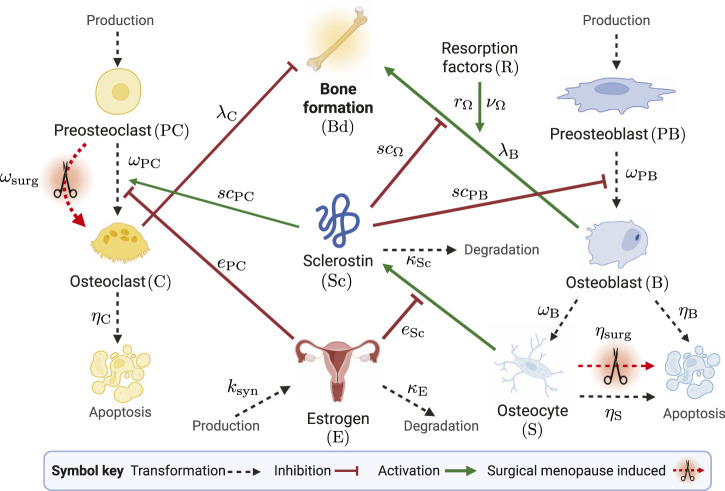
Schematic of mathematical model species in the bone remodeling process. The transformations of cells through differentiation, production, and degradation are illustrated by black dashed arrows. Signaling-related inhibition interactions are shown by red flat-head arrows, and activation interactions are shown as green solid arrows. Parameters that govern interactions are shown near the corresponding arrows. The schematic shows estrogen as an inhibitor of osteoclast differentiation and sclerostin production. Surgical menopause-induced changes are depicted by red dashed arrows with scissors icons. Created with BioRender.com.

We now describe our model that extends the work of Jörg et al. (2022) to account for surgical menopause. To explore the impact of surgical menopause on bone remodeling, we incorporate two new mechanisms into the model: increased apoptosis of osteocytes through a term η(t) and increased differentiation of osteoclasts through a term ω(t) . We include these effects through apoptosis and differentiation rates, which depend on the time since surgical menopause. This time dependence captures inflammation and metabolic responses outside the BMU. In Figure 2, we present a schematic of the model, illustrating how different cells and chemical signals interact and influence bone formation. The mechanisms affected by estrogen are shown in Figure 2, with new surgical menopause effects highlighted by red dashed arrows with scissor icons, representing surgical removal of the ovaries.

Each cell population and chemical concentration is scaled by reference values for ease of computation: PC and C are scaled by the number of preosteoclasts produced per day in the BMU; PB , B , S , and Sc are scaled relative to the number of preosteoblasts produced per day; and estrogen is scaled by the initial concentration of estrogen at the onset of menopause.

Activation and repression signaling interactions are modeled with saturating Hill-type functions:

$$f^{+}\left(X,x_{i}\right)=\frac{X}{X+x_{i}},\;f^{-}\left(X,x_{i}\right)=\frac{x_{i}}{X+x_{i}},$$ (1)

respectively. For each type of interaction by species X , the threshold concentration where half of the interaction strength is achieved is the parameter $x_{i}$ , where i denotes the target of the signaling interaction.

For simplicity, we assume estrogen is described by an algebraic equation, and its form depends on the type of menopause investigated. The normalized estrogen concentration over time during natural menopause is (Jörg et al., 2022):

$$E_{\text{nat}}\left(t\right)=\left\{\begin{array}{cc} 1,\; & t\leq t_{m}\\ \frac{1}{1+\left(t-t_{m}\right)/\tau_{\text{E}}},\; & t>t_{m}, \end{array}\right.$$ (2)

where $t_{m}$ is the time of onset of estrogen decline and $\tau_{\text{E}}$ is the characteristic time of estrogen decline. We capture the sudden and rapid decrease in relative estrogen concentration due to oophorectomy surgery using the following equation, which is derived assuming estrogen is still synthesized at a reduced constant (zero-order) rate of $k_{\text{syn}}$ post-surgery and degraded at a first-order rate with rate constant $\kappa_{\text{E}}$ :

$$E_{\text{surg}}\left(t\right)=\left\{\begin{array}{cc} 1,\; & t\leq t_{m}\\ \left(1-\frac{k_{\text{syn}}}{\kappa_{\text{E}}}\right)\exp\left(-\kappa_{\text{E}}\left(t-t_{m}\right)\right)+\frac{k_{\text{syn}}}{\kappa_{\text{E}}},\; & t>t_{m}, \end{array}\right.$$ (3)

where the degradation rate of estrogen is defined as $\kappa_{\text{E}}=\ln\left(2\right)/t_{1\text{/}2}$ and $t_{1\text{/}2}$ is the half-life of estrogen, which is 161 min in postmenopausal women (Ginsburg et al., 1998). The initial concentration of estrogen before surgery is 156 pg/mL, and the estrogen level stabilizes and reaches a steady state by about 30 days post-surgery to an average value of 15 pg/mL (Bellanti et al., 2013). Thus, we set the post-surgery normalized estrogen concentration as $E_{\text{surg}}\left(t_{m}+30days\right)=\frac{15\text{ pg/mL}}{156\text{ pg/mL}}$ . The synthesis rate after surgery is calculated as $k_{\text{syn}}=\kappa_{\text{E}}E_{\text{surg}}\left(t_{m}+30days\right)$ . Figure 1c shows the difference in the estrogen decrease in the case of natural menopause compared to surgical menopause, described by Equations 2, 3, where the inset illustrates the timescale of rapid estrogen decline in surgical menopause.

The changes in cell number over time of the preosteoclasts (PC) and osteoclasts (C) are given by

$$\frac{dPC}{dt}=1-\omega \left(t\right)f^{-}\left(E,e_{\text{PC}}\right)f^{+}\left(Sc,sc_{\text{PC}}\right)PC$$ (4)

and

$$\frac{dC}{dt}=\omega \left(t\right)f^{-}\left(E,e_{\text{PC}}\right)f^{+}\left(Sc,sc_{\text{PC}}\right)PC-\eta_{\text{C}}C,$$ (5)

respectively. Preosteoclasts are produced at a constant basal rate of one upon scaling and differentiate into osteoclasts at a rate of ω(t) . The presence of estrogen inhibits osteoclast differentiation, while sclerostin activates this differentiation with thresholds $e_{\text{PC}}$ and $sc_{\text{PC}}$ , respectively. This effect of sclerostin captures the role of RANKL implicitly. Osteoclast apoptosis occurs at a rate $\eta_{\text{C}}$ . Note that we do not include the apoptosis of any precursor cells (preosteoclasts or preosteoblasts) or any effect of estrogen on osteoclast apoptosis, as these were estimated to have a negligible impact in Jörg et al. (2022).

The first effect of surgical menopause is included in a new time-dependent differentiation rate for preosteoclasts to osteoclasts, ω(t) , defined as

$$\omega \left(t\right)=\left\{\begin{array}{cc} \omega_{\text{PC}},\; & t\leq t_{m},\\ \omega_{\text{PC}}\left(1+\omega_{\text{surg}}\exp\left(-\tau \left(t-t_{m}\right)\right)\right),\; & t>t_{m}. \end{array}\right.$$ (6)

Before estrogen decline $t\leq t_{m}$ and for natural menopause according to the Jörg et al. (2022) model, differentiation occurs with a constant rate $\omega_{\text{PC}}$ . At the onset of rapidly decreased estrogen due to surgical menopause (Equation 3; Figure 1c), the differentiation rate increases by $\omega_{\text{surg}}$ , e.g., for a 10% increase, then $\omega_{\text{surg}}=0.1$ . This increased rate of differentiation lasts for a period defined by the parameter τ , such that longer-lasting effects of surgery are defined by a smaller τ .

The preosteoblast (PB) and osteoblasts (B) populations are governed by

$$\frac{dPB}{dt}=1-\omega_{\text{PB}}f^{-}\left(Sc,sc_{\text{PB}}\right)PB$$ (7)

and

$$\frac{dB}{dt}=\omega_{\text{PB}}f^{-}\left(Sc,sc_{\text{PB}}\right)PB-\left(\eta_{\text{B}}+\omega_{\text{B}}\right)B,$$ (8)

respectively. Preosteoblasts are produced at a constant basal rate of one upon scaling and differentiate into osteoblasts at a rate of $\omega_{\text{PB}}$ inhibited by sclerostin, capturing the role of Wnt implicitly. Osteoblasts have an apoptosis rate of $\eta_{\text{B}}$ and are further differentiated into osteocytes at a rate of $\omega_{\text{B}}$ .

The dynamic population of osteocytes (S) is described by

$$\frac{dS}{dt}=\omega_{\text{B}}B-\eta \left(t\right)S.$$ (9)

Osteocytes are derived from osteoblasts and are removed at a rate dependent on η(t) .

The second effect of surgical menopause is increased apoptosis of osteocytes via the new time-dependent rate, η(t) , defined as

$$\eta \left(t\right)=\left\{\begin{array}{cc} \eta_{\text{S}},\; & t\leq t_{m},\\ \eta_{\text{S}}\left(1+\eta_{\text{surg}}\exp\left(-\tau \left(t-t_{m}\right)\right)\right),\; & t>t_{m}. \end{array}\right.$$ (10)

Similarly to Equation 6, we assume that before menopause and for natural menopause according to the Jörg et al. (2022) model, differentiation occurs at a constant rate of $\eta_{\text{S}}$ . At surgical menopause onset, apoptosis increases by $\eta_{\text{surg}}$ , then returns to previous levels over a timescale of τ . This timescale is assumed to be equal to the timescale of increased osteoclast differentiation in Equation 6.

The production of the signaling molecule sclerostin is governed by

$$\frac{dSc}{dt}=f^{-}\left(E,e_{\text{Sc}}\right)S-\kappa_{\text{Sc}}Sc.$$ (11)

where sclerostin is produced by osteocytes at a rate inhibited by estrogen with threshold $e_{\text{Sc}}$ and is degraded at rate $\kappa_{\text{Sc}}$ . Sclerostin affects bone formation through activation of osteoclastogenesis in Equations 4, 5 and inhibition of osteoblastogenesis in Equations 7, 8, respectively.

Bone density, Bd , is determined by

$$\frac{dBd}{dt}=\lambda_{\text{B}}Bf^{-}\left(Sc,sc_{\Omega }\right)\left(1+\nu_{\Omega }f^{+}\left(R,r_{\Omega }\right)\right)-\lambda_{\text{C}}C,$$ (12)

where the resorption factor R is assumed to be equal to the amount of osteoclasts present, i.e., R=C . Osteoclasts inhibit bone formation, and osteoblasts contribute to bone formation. From Equation 12, the rate of bone density change increases proportionally to osteoblast number at a rate of $\lambda_{\text{B}}$ . This production is modulated by sclerostin inhibition with a threshold of $sc_{\Omega }$ and resorption factor R activation with a strength of $\nu_{\Omega }$ and a threshold of $r_{\Omega }$ . Bone density decreases through bone resorption by osteoclasts at a rate $\lambda_{\text{C}}$ . To determine BMD, we scale bone density by bone mineral content, which is a constant ${BMC}_{0}$ , so we have $BMD={BMC}_{0}Bd$ .

To summarize, our model extensions introduce three new parameters fit to data: the factor for peak increase in osteocyte apoptosis due to surgery $\left(\eta_{\text{surg}}\right)$ , the factor for peak increase in osteoclast differentiation due to surgery $\left(\omega_{\text{surg}}\right)$ , and the timescale during which these effects last (τ) . The model parameters and species definitions for natural menopause are listed in Table 3, the initial conditions for the variables are listed in Table 2, and the model parameters and species definitions for surgical menopause are listed in Table 4.

**TABLE 2:** Initial conditions taken at steady state at 30 years before menopause onset. All variables are in dimensionless form.

Variable	Meaning	Initial value
PB	Preosteoblasts	$2.16\times 10^{2}$
PC	Preosteoclasts	$4.10\times 10^{3}$
C	Osteoclasts	$4.20\times 10^{1}$
B	Osteoblasts	$1.07\times 10^{2}$
S	Osteocytes	$6.12\times 10^{2}$
Sc	Sclerostin	$1.12\times 10^{4}$
Bd	Bone density	1

### 2.3 Model solution and parameter estimation

We are interested in understanding and parameterizing the impacts of natural and surgical menopause on the dynamics of bone mineral density, Bd . Therefore, we solve the model ODEs and algebraic equations defined in Equations 1–12 using ode45 in MATLAB, with absolute and relative tolerances as $10^{-8}$ , from 30 years before menopause onset to 30 years after menopause onset. Thus, the initial condition is 30 years before menopause onset, whether natural or surgical. For each simulation, we initialize the dynamic species based on their steady-state values at the initial condition. The Jörg et al. (2022) model includes premenopausal BMD decline. To determine the steady state solutions to Equations 4, 5, 7–9, 11 while Bd is fixed at a value of 1, we solve the system of equations with time derivatives set to 0 using the fsolve function in MATLAB. The other species do not depend on Bd . This initialization is called within the parameter estimation routine for the natural menopause case, as the fitted parameters affect the initial conditions. These updated values are reported in Table 2.

To estimate parameters in both the natural and surgical menopause mechanisms, we use the lsqnonlin function in MATLAB, which solves the nonlinear least-square objective function in Equation 13 using the Levenberg-Marquardt algorithm (Levenberg, 1944; Marquardt, 1963):

$${\mathrm{arg min}}_{\text{q}}\underset{j}{\sum }{\left[{BMD}_{\text{data}}\left(t_{j}\right)-BMD\left(t_{j},q\right)\right]}^{2}.$$ (13)

Here, ${BMD}_{\text{data}}\left(t_{j}\right)$ is the natural BMD data at measurement times $t_{j}$ , and $BMD\left(t_{j},q\right)$ is the BMD predicted by the model at the same times using parameters q . For each parameter estimation procedure, lsqnonlin algorithm options are set to tolerances of $10^{-8}$ and a maximum of 10,000 function evaluations and iterations. We use MATLAB version R2024b in Windows on a PC with 11th Generation Intel Core i7-11700 8 Core processor. We have provided model code and files in a repository at https://github.com/ashleefv/ SurgicalMenopauseBone (Nelson et al., 2025b).

With the natural menopause BMD data described in Section 2.1, we first aim to re-estimate model parameters related to the impact of estrogen on osteoclastogenesis and the production of sclerostin by osteocytes for natural menopause. This corresponds to parameters $q_{\text{NM}}=\left\{e_{\text{PC}},e_{\text{Sc}}\right\}$ , and the estimated values of these parameters are listed in Table 3. With the estimated $q_{\text{NM}}$ , we then use surgical menopause BMD data to estimate surgical menopause parameters $q_{\text{SM}}=\left\{\eta_{\text{surg}},\tau ,\omega_{\text{surg}}\right\}$ in the 15 years (short-term) and in the 30 years (long-term) after onset of menopause due to surgery. For the surgical menopause cases, upper and lower bounds are used to constrain the parameter space. The lower bounds are $q_{\text{SM}}=\left\{0,0,0\right\}$ , which signify no effect, permanent effect, and no effect, respectively. The upper bounds are more subjective and were selected to yield only reasonable responses; we would need cell population data to further interrogate these with different upper bounds. The upper bounds used are $q_{\text{SM}}=\left\{5,1/365,5\right\}$ , signifying that the peaks of η(t) and ω(t) at menopause onset are 5 + 1 = 6 times higher than baseline values in natural menopause and the surgery induced effects last a minimum of 1 day. The estimated values of these parameters are listed in Table 4.

**TABLE 3:** Natural menopause (NM) model parameters with parameters taken from Jörg et al. (2022) or estimated using the procedures outlined in Section 2.3. ND: no dimensions.

Parameter	Meaning	Value	Dimension	Source
$\omega_{\text{PC}}$	Differentiation rate of PC→C	0.93	${\text{day}}^{-1}$	Jörg et al. (2022)
$\omega_{\text{PB}}$	Differentiation rate of PB→B	0.32	${\text{day}}^{-1}$	Jörg et al. (2022)
$\omega_{\text{B}}$	Differentiation rate of B→S	$6.4\times 10^{-4}$	${\text{day}}^{-1}$	Jörg et al. (2022)
$\eta_{\text{B}}$	Apoptosis rate of B	$8.678\times 10^{-3}$	${\text{day}}^{-1}$	Jörg et al. (2022)
$\eta_{\text{C}}$	Apoptosis rate of C	$1.096\times 10^{-4}$	${\text{day}}^{-1}$	Jörg et al. (2022)
$\eta_{\text{S}}$	Apoptosis rate of S	$1.1\times 10^{-4}$	${\text{day}}^{-1}$	Jörg et al. (2022)
$\lambda_{\text{B}}$	First-order bone formation rate	$1.29\times 10^{-6}$	${\text{day}}^{-1}$	Jörg et al. (2022)
$\lambda_{\text{C}}$	First-order bone resorption rate	$3.82\times 10^{-6}$	${\text{day}}^{-1}$	Jörg et al. (2022)
$\kappa_{\text{Sc}}$	Degradation rate of Sc	0.05	${\text{day}}^{-1}$	Jörg et al. (2022)
$\tau_{\text{E}}$	Characteristic time of estrogen decline	2.6	year	Jörg et al. (2022)
$e_{\text{PC}}$	Threshold for estrogen inhibition of PC→C	0.2556	ND	Estimated, NM
$e_{\text{Sc}}$	Threshold for estrogen inhibition of Sc production	10.59	ND	Estimated, NM
$sc_{\text{PC}}$	Threshold for sclerostin activation of PC→C	$8.6\times 10^{6}$	ND	Jörg et al. (2022)
$sc_{\text{PB}}$	Threshold for sclerostin inhibition of PB→B	$1.63\times 10^{2}$	ND	Jörg et al. (2022)
$sc_{\Omega }$	Threshold for sclerostin inhibition of Bd	$3.04\times 10^{3}$	ND	Jörg et al. (2022)
$r_{\Omega }$	Threshold for resorption activation of Bd	$1.02\times 10^{3}$	ND	Jörg et al. (2022)
${BMC}_{0}$	Steady-state BMC	0.8	ND	Jörg et al. (2022)
$\nu_{\Omega }$	Maximum relative effect of resorption	$1.08\times 10^{3}$	ND	Jörg et al. (2022)

**TABLE 4:** Surgical menopause (SM) model parameters from sources, calculated as described in Section 2.2, and estimated using the procedures outlined in Section 2.3 for short-term (15 years or less post-surgery) and long-term (up to 30 years post-surgery) data. ND: no dimensions.

Parameter	Meaning	Value	Dimension	Source
$\kappa_{\text{E}}$	Estrogen degradation rate post-surgery	6.1996	${\text{day}}^{-1}$	Ginsburg et al. (1998)
$k_{\text{syn}}$	Estrogen synthesis rate post-surgery	0.6	${\text{day}}^{-1}$	Calculated
$E_{\text{surg}}\left(t_{m}+30\right)$	Estrogen concentration 30 days post-surgery	0.096	ND	Bellanti et al. (2013)
$\eta_{\text{surg}}$	Apoptosis of S post-surgery (short-term)	5	ND	Estimated, SM short-term
τ	Timescale of post-surgery dynamics (short-term)	$9.7\times 10^{-3}$	day^-1^	Estimated, SM short-term
$\omega_{\text{surg}}$	Enhanced C differentiation post-surgery (short-term)	1.86	ND	Estimated, SM short-term
$\eta_{\text{surg}}$	Apoptosis of S post-surgery (long-term)	0.4174	ND	Estimated, SM long-term
τ	Timescale of post-surgery dynamics (long-term)	0	${\text{day}}^{-1}$	Estimated, SM long-term
$\omega_{\text{surg}}$	Enhanced C differentiation post-surgery (long-term)	0.2155	ND	Estimated, SM long-term

We calculate sensitivity by varying the surgical menopause model parameters $q_{\text{SM}}=\left\{\eta_{\text{surg}},\tau ,\omega_{\text{surg}}\right\}$ by 25% relative to the best-fit parameters. For instance, the upper bound is calculated using a 25% increase in $\eta_{\text{surg}}$ and $\omega_{\text{surg}}$ and a 25% decrease in τ , which corresponds to longer-lasting effects of surgery.

**FIGURE 3:**
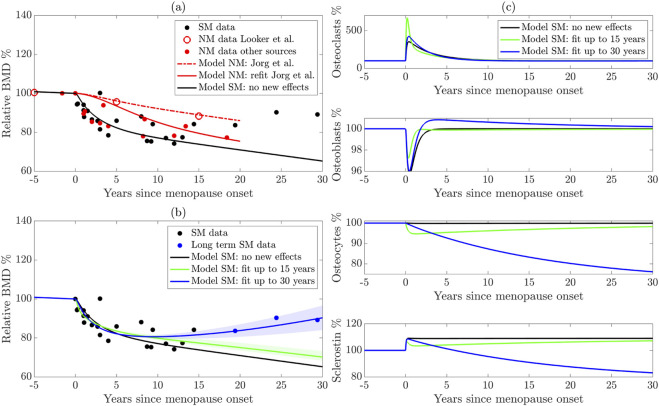
**(a)** Reparameterization of the Jörg et al. (2022) model using more sources of natural menopause (NM) data. The dashed red curve shows the original model, the solid red curve shows the new parameterization, and the black solid curve shows the model with sudden estrogen loss alone, without any new effects. **(b)** Extension of the model to surgical menopause. New effects are parameterized using long-term (blue curve) and short-term (green curve) data. The shaded regions highlight model sensitivity to parameters. **(c)** Cell populations from the surgical menopause model using the three parameter sets shown in panel **(b)**. NM: Natural menopause. SM: surgical menopause.

**FIGURE 4:**
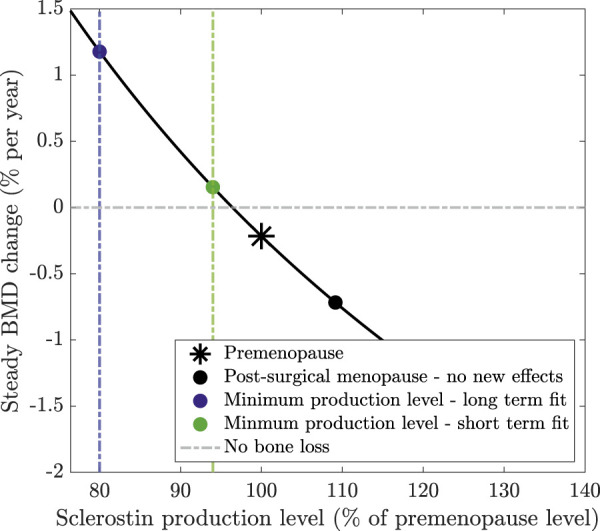
Steady BMD change as a function of sclerostin production levels at steady state (relative to the premenopause level). Steady-state cell concentrations are calculated assuming that no new effects are included in the model. Sclerostin production is then adjusted by a percentage of its premenopause level to calculate the resulting steady-state change in BMD.

We determine the BMD sensitivity to sclerostin levels presented in Section 3.3 by calculating the steady state levels of cells and chemical concentrations, defined by Equations 4–11. We then calculate the constant rates of BMD loss or production using Equation 12 with the steady-state values of osteocytes, sclerostin, and osteoblasts. Estrogen is assumed to be at a fixed value either at premenopause levels of E=1 or at the post-surgical menopause levels of $E=k_{\text{syn}}/\kappa_{\text{E}}$ . We calculate the curve of steady BMD change rates in Figure 4 using the model with no new effects, namely, with $\eta_{\text{surg}}=\omega_{\text{surg}}=0$ . We perturb the sclerostin production rate by adding a control parameter α multiplying sclerostin production in Equation 11 so that its steady state is defined by $Sc=\alpha f^{-}\left(E,e_{\text{Sc}}\right)S/\kappa_{\text{Sc}}$ . The curve is defined by α∈[0.7,1.2] with $E=k_{\text{syn}}/\kappa_{\text{E}}$ . The reference points for the long and short term model fits in Figure 4, are defined by α=0.8 and α=0.94 , respectively, reflecting that the osteocyte levels in each case obtain minimum values of 80% and 94% of the premenopause levels in our results (Figure 3).

## 3 Results

Using the mathematical framework introduced in Section 2.2, we study how mechanisms mediated by estrogen loss affect bone remodeling in both natural and surgical menopause. We focus on how BMD is affected decades after menopause and compare the BMD dynamics to both natural and surgical menopause data described in Section 2.1. We use data from natural and surgical menopause patients (Figure 1) and the mathematical models outlined in Section 2.2 with the methods described in Section 2.3 to parameterize the models and compare their results with the relevant data. Using new parameters and mechanisms, we identify key pathways that drive BMD decline and rebound and propose new treatment directions based on our results.

### 3.1 Parameterized model of natural menopause captures BMD behavior in larger lumbar spine dataset

To assess the model output, we compare the model-predicted BMD with the experimental data described in Section 2.1. In Figure 3a for the natural menopause case, we present the average BMD for natural menopause patients in red markers for the larger dataset and distinguish the femur BMD data (Looker et al., 1998) that were used to parameterize the Jörg et al. (2022) model mathematical model with open red markers. The Jörg et al. (2022) model results (red dashed curve) fit the Looker et al. (1998) data well but do not fit the additional natural menopause data measured in the lumbar spine, which show a faster BMD decline.

To improve the model’s accuracy in predicting lumbar spine BMD dynamics after menopause, we estimate key parameters associated with natural menopause using aggregated natural menopause data. In particular, we estimate functional thresholds that depend on estrogen in both preosteoclast to osteoclast differentiation and sclerostin production from osteocytes, which are $e_{\text{PC}}$ and $e_{\text{Sc}}$ , respectively. Our reparameterized model of natural menopause (shown as a red solid curve) captures this rapid BMD decrease, and this behavior is within the experimental error (Figure 3a). We terminate natural menopause simulations at approximately 20 years post-menopause onset, matching the time period of our dataset.

### 3.2 With new mechanisms, model of surgical menopause reproduces BMD trends in both short- and long-term data

Figure 3b shows the average relative BMD of surgical menopause patients 15 years post-surgery (black markers) and long-term data beyond 15 years (blue markers), indicating a rebound in BMD. Note that these are the same data in Figure 1b, where the error bars are shown.

To illustrate that new mechanisms are necessary to capture the surgical menopause behavior, we first show the model dynamics (Figures 3a,b black curve) using newly estimated bone parameters for natural menopause from Section 3.1 and the estrogen decline in surgical menopause from Equation 3 without including any new effects on cell dynamics, i.e., $\eta_{\text{surg}}=0$ and $\omega_{\text{surg}}=0$ . The effect of sudden estrogen loss alone enhances BMD loss compared to natural estrogen decline in Figure 3a. Indeed, the model with no new cell dynamics closely matches the surgical menopause data for the first 1–3 years post-surgery but overpredicts the extent of BMD loss in later years (Figures 3a,b black curve). This result suggests that surgical menopause involves both an increased rate of bone loss in the short term and a subsequent slowing of bone loss in the long term, which is not captured by simply including the onset of a sudden estrogen decline in the natural menopause model of Jörg et al. (2022).

We estimate the new parameters modulated by surgical menopause in our model (Table 4): the percentage increase osteocyte apoptosis rate $\left(\eta_{\text{surg}}\right)$ , the increased differentiation rate of preosteoclasts $\left(\omega_{\text{surg}}\right)$ , and the timescale over which the effects occur (τ) , using data in Figure 1b. We obtain root mean squared errors in BMD prediction of 4.59% and 4.63% compared to the surgical menopause data up to 15 years and 30 years, respectively. Using the short and long time scale datasets resulted in different long-term BMD dynamics (Figure 3b). The short-term surgical menopause model better captures the data for the 15-year period compared to the case without new effects (Figures 3a,b black curve). The long-term surgical menopause model also fits these data well and has a BMD rebound not observed in the short-term case. The sensitivity of the model predictions to the parameter values is shown by the corresponding shaded area around each curve. Across these sensitivity regions, the model’s behavior over the 2 years post-surgery is insensitive to the new effects, whereas parameter variations substantially alter its long-term predictions.

To understand other differences in these parameterized surgical menopause models, we show the dynamics of the osteoclast, osteoblast, and osteocyte cell populations as well as the levels of sclerostin after menopause (Figure 3c). The no-new-effects case and the short-term case show that all species (except sclerostin) return to approximately their premenopausal levels within 10 years. This is expected because the timescale of surgical effects, τ , is small for the short-term model. The short-term effect is observed in the osteoclast (osteocyte) population, with a sharp increase (decrease) at the onset of menopause, followed by a rebound. The sudden increase in osteoclast population yields steeper dips in Figure 3b, i.e., relative BMD in the short-term surgical menopause model is lower than other models immediately after menopause onset.

In the long-term surgical menopause case, we see a smaller increase in osteoclastogenesis after menopause onset compared to the short-term case; $\omega_{\text{surg}}$ is an order of magnitude smaller in the long-term case than the short-term case. Osteocyte density also decreases more slowly in the long-term surgical menopause model, but continues after the onset of menopause; τ=0 for long-term surgical menopause effects, making the impact of surgery permanent. Interestingly, the percentage of osteoblasts decreases after surgery, then rebounds to a slightly higher level for an extended period.

### 3.3 Osteocyte and sclerostin dynamics are key drivers of slowing BMD loss

In our model, the signalling molecule sclerostin is produced solely by osteocytes, with its production rate increasing as estrogen levels fall. Sclerostin levels play a crucial role in regulating bone formation and resorption. Figure 4 shows the sensitivity of BMD production rate as a function of the sclerostin production rate (method detailed in Section 2.3). Higher sclerostin production rates lead to increased bone loss, whereas sclerostin production rates below 96% of the premenopause level lead to bone formation. In the absence of additional modeling effects, the production rate of sclerostin postmenopause is 10% higher than premenopause (Figure 3c). This leads to a bone loss of −0.75% per year postmenopause compared to −0.22% per year premenopause (marked on Figure 4).

Our model extension, which simulates a surgically induced temporary increase in osteocyte apoptosis, reduces sclerostin production, slows bone loss, and captures the reduced bone loss or rebound observed in clinical data. The long-term surgical menopause model yields a continually declining osteocyte population with osteocyte levels reaching ≈ 80% of their premenopause levels 25 years post-surgery. In the short-term model, the osteocyte population reaches ≈ 94% of its premenopause levels before slowly returning to 100% . If an 80% or 94% lower level of osteocytes persisted at steady state, this would yield BMD growth of 1.2% or 0.15% per year, respectively, as marked on Figure 4. Although these osteocyte levels are not obtained at steady state because the bone system is still responding to surgery, this explains how these osteocyte values alter sclerostin production and, subsequently, BMD, resulting in a reduced rate of bone loss or even a rebound in BMD.

## 4 Discussion

We present a mathematical model of bone remodeling to quantify the effects of estrogen loss in surgical menopause. Since experimental data suggest that surgical and natural menopause affect different mechanisms of bone remodeling, we extended an existing mathematical model of bone remodeling to incorporate increased osteoclast differentiation and osteocyte apoptosis. The objective of this framework is to understand and capture trends seen in newly aggregated physiological data: (1) surgical menopause leads to an increased loss of BMD in the short term, and (2) this loss slows or even rebounds by 10 or more years post-surgery.

The BMD predictions after reparameterization to the larger natural menopause BMD dataset better capture the overall trends of the natural menopause data compared to the previous mathematical model (Jörg et al., 2022). These parameters, $e_{\text{PC}}$ and $e_{\text{Sc}}$ , influence the strength of estrogen signaling on osteoclastogenesis and sclerostin release by osteocytes. Compared to the previous mathematical model, our parameter fitting results in smaller threshold values for the estrogen signaling pathway related to osteoclastogenesis, resulting in more osteoclast differentiation for a higher concentration of estrogen. These parameter changes capture the overall larger decrease in BMD shown in the newly aggregated data.

Our new surgical menopause model that incorporates mechanisms impacted by the sudden loss of estrogen and inflammation resulting from surgery, including increases in osteocyte apoptosis and osteoclastogenesis follows the sharp decrease in BMD in the first 15 years post-surgery and a rebound in BMD 15–30 years post-surgery, consistent with the clinical data. To understand which mechanisms underlie this varied behavior, we fit our surgical menopause model to two datasets: BMD data from up to 15 years and up to 30 years post-surgery. The short-term data fit indicates a higher osteocyte apoptosis rate and a lower osteoclast differentiation rate compared to the long-term data fit. Osteocyte levels differ substantially between the long-term and short-term model calibrations, with the long-term parameters yielding slower but permanent rates of osteocyte apoptosis due to surgical menopause. These results show that the model is capable of fitting the available data, but long-term predictions should be made after the model is trained on long-term data.

Our mathematical study does have some limitations. Here, we do not consider the mechanical mechanisms underlying the initiation and regulation of bone remodeling, which are mediated by osteocytes’ response to mechanical strain (Bonewald and Johnson, 2008; Santos et al., 2009). However, sclerostin levels have been shown to decrease with mechanical loading (Robling et al., 2008), so another potential mechanism for decreasing sclerostin could be to incorporate strain into our mathematical framework. We model bone remodeling within an individual BMU and do not account for strain-induced deformation of the bone matrix. This work also does not incorporate hormonal interventions or bone remodeling treatments. Most osteoporosis treatments are classified as anti-resorptive agents that inhibit osteoclast-mediated resorption to prevent further bone loss, but they can cause side effects. For example, bisphosphonates are widely prescribed antiresorptive treatments to promote osteoclast apoptosis (Rodan and Fleisch, 1996; Rogers et al., 1999; Russell et al., 1999; Berkhout et al., 2015), but they can lose efficacy over time and lead to osteonecrosis and atypical femoral fractures (Shane et al., 2014; Khosla et al., 2007; Whitaker et al., 2012). Another anti-resorptive treatment is the anti-RANKL monoclonal antibody denosumab, which inhibits osteoclastogenesis (Khosla and Hofbauer, 2017). Other antibody treatments targeting sclerostin produced from osteocytes show promising results of increased bone formation and decreased bone resorption in rodent models (Li et al., 2009; Zhang et al., 2016; Tian et al., 2011) and in postmenopausal women with bone loss (McClung et al., 2014; Recker et al., 2015; Cosman et al., 2016; Zhang et al., 2016; Chavassieux et al., 2019; McClung, 2017). However, bone formation only lasts a few months (Ominsky et al., 2017). Therefore, treatment with sclerostin inhibitors is only recommended for 6–12 months (McClung, 2017). Our parameter estimates indicate promising avenues for treatment in patients undergoing surgical menopause, suggesting that targeting osteocyte or osteoblast dynamics may support long-term BMD preservation. Implementing treatment methods on this system requires further investigation.

## Data Availability

The original contributions presented in the study are included in the article, further inquiries can be directed to the corresponding author.

## References

[B1] Aitken J. M. Hart D. M. Anderson J. B. Lindsay R. Smith D. A. Speirs C. F. (1973). Osteoporosis after oophorectomy for non-malignant disease in premenopausal women. Br. Med. J. 2, 325–328. 10.1136/bmj.2.5862.325 4704517 PMC1589320

[B2] Allen M. R. Burr D. B. (2014). “Bone modeling and remodeling,” in Basic and applied bone biology. Editors Burr D. B. Allen M. R. (New York: Academic Press), 75–90. 10.1016/B978-0-12-416015-6.00004-6

[B3] Almeida M. Martin-Millan M. Plotkin L. I. Stewart S. A. Roberson P. K. Kousteni S. (2007). Skeletal involution by age-associated oxidative stress and its acceleration by loss of sex steroids. J. Biol. Chem. 282, 27285–27297. 10.1074/jbc.M702810200 17623659 PMC3119455

[B4] Atkins G. J. Rowe P. S. Lim H. P. Welldon K. J. Ormsby R. Wijenayaka A. R. (2011). Sclerostin is a locally acting regulator of late-osteoblast/preosteocyte differentiation and regulates mineralization through a MEPE-ASARM-dependent mechanism. J. Bone Mineral Res. 26, 1425–1436. 10.1002/jbmr.345 21312267 PMC3358926

[B5] Bellanti F. Matteo M. Rollo T. De Rosario F. Greco P. Vendemiale G. (2013). Sex hormones modulate circulating antioxidant enzymes: impact of estrogen therapy. Redox Biol. 1, 340–346. 10.1016/j.redox.2013.05.003 24024169 PMC3757703

[B6] Bellido T. (2014). Osteocyte-driven bone remodeling. Calcif. Tissue Int. 94, 25–34. 10.1007/.s00223-013-9774-y 24002178 PMC3947228

[B7] Berkhout J. Stone J. A. Verhamme K. M. Stricker B. H. Sturkenboom M. C. Danhof M. (2015). Application of a systems pharmacology-based placebo population model to analyze long-term data of postmenopausal osteoporosis. CPT Pharmacometrics and Syst. Pharmacol. 4, 516–526. 10.1002/psp4.12006 26451331 PMC4592531

[B8] Berkhout J. Stone J. A. Verhamme K. M. Danhof M. Post T. M. (2016). Disease systems analysis of bone mineral density and bone turnover markers in response to alendronate, placebo, and washout in postmenopausal women. CPT Pharmacometrics and Syst. Pharmacol. 5, 656–664. 10.1002/psp4.12135 27869358 PMC5193000

[B9] Bonewald L. F. Johnson M. L. (2008). Osteocytes, mechanosensing and wnt signaling. Bone 42, 606–615. 10.1016/.j.bone.2007.12.224 18280232 PMC2349095

[B10] Brennan O. Kennedy O. D. Lee T. C. Rackard S. M. O’Brien F. J. (2011). Effects of estrogen deficiency and bisphosphonate therapy on osteocyte viability and microdamage accumulation in an ovine model of osteoporosis. J. Orthop. Res. 29, 419–424. 10.1002/jor.21229 20886644

[B11] Brennan M. A. Haugh M. G. O’Brien F. J. McNamara L. M. (2014). Estrogen withdrawal from osteoblasts and osteocytes causes increased mineralization and apoptosis. Hormone Metabolic Res. 46, 537–545. 10.1055/.s-0033-1363265 24446157

[B12] Chaiya I. Rattanakul C. (2017). “An impulsive mathematical model of bone formation and resorption: effects of parathyroid hormone, calcitonin and impulsive estrogen supplement,” in *Advances in Difference Equations* 2017, 153. 10.1186/s13662-017-1206-2

[B13] Challberg J. Ashcroft L. Lalloo F. Eckersley B. Clayton R. Hopwood P. (2011). Menopausal symptoms and bone health in women undertaking risk reducing bilateral salpingo-oophorectomy: significant bone health issues in those not taking HRT. Br. J. Cancer 105, 22–27. 10.1038/bjc.2011.202 21654687 PMC3137416

[B14] Chavassieux P. Chapurlat R. Portero-Muzy N. Garcia P. Brown J. P. Libanati C. (2019). Bone-forming and antiresorptive effects of romosozumab in postmenopausal women with osteoporosis: bone histomorphometry and microcomputed tomography analysis after 2 and 12 months of treatment. J. Bone Mineral Res. 34, 1597–1608. 10.1002/jbmr.3735 31233639 PMC7027577

[B15] Chittacharoen A. Theppisai U. Sirisriro R. (1999). Bone mineral density in natural and surgically-induced menopause. Int. J. Gynecol. and Obstetrics 66, 193–194. 10.1016/S0020-7292(99)00049-1 10468352

[B16] Choi B. G. Vilahur G. Cardoso L. Fritton J. C. Ibanez B. Zafar M. U. (2008). Ovariectomy increases vascular calcification *via* the OPG/RANKL cytokine signalling pathway. Eur. J. Clin. Investigation 38, 211–217. 10.1111/j.1365-2362.2008.01930.x 18279396 PMC4811027

[B17] Cohen J. V. Chiel L. Boghossian L. Jones M. Stopfer J. E. Powers J. (2012). Non-cancer endpoints in BRCA1/2 carriers after risk-reducing salpingo-oophorectomy. Fam. Cancer 11, 69–75. 10.1007/s10689-011-9480-8 21898151

[B18] Cook C. V. Islam M. A. Smith B. J. Ford Versypt A. N. (2022). Mathematical modeling of the effects of Wnt-10b on bone metabolism. AIChE J. 68, e17809. 10.1002/aic.17809 36567819 PMC9788157

[B19] Cook C. V. Lighty A. M. Smith B. J. Ford Versypt A. N. (2024). A review of mathematical modeling of bone remodeling from a systems biology perspective. Front. Syst. Biol. 4, 1368555. 10.3389/fsysb.2024.1368555 40012834 PMC11864782

[B20] Cosman F. Crittenden D. B. Adachi J. D. Binkley N. Czerwinski E. Ferrari S. (2016). Romosozumab treatment in postmenopausal women with osteoporosis. N. Engl. J. Med. 375, 1532–1543. 10.1056/NEJMoa1607948 27641143

[B21] Creecy A. Damrath J. G. Wallace J. M. (2021). Control of bone matrix properties by osteocytes. Front. Endocrinol. 11, 578477. 10.3389/fendo.2020.578477 33537002 PMC7848033

[B22] Delgado-Calle J. Bellido T. (2022). The osteocyte as a signaling cell. Physiol. Rev. 102, 379–410. 10.1152/.physrev.00043.2020 34337974 PMC8858675

[B23] Delgado-Calle J. Sato A. Y. Bellido T. (2017). Role and mechanism of action of sclerostin in bone. Bone 96, 29–37. 10.1016/j.bone.2016.10.007 27742498 PMC5328835

[B24] Emerton K. B. Hu B. Woo A. A. Sinofsky A. Hernandez C. Majeska R. J. (2010). Osteocyte apoptosis and control of bone resorption following ovariectomy in mice. Bone 46, 577–583. 10.1016/j.bone.2009.11.006 19925896 PMC2824001

[B25] Fakkert I. E. Teixeira N. Abma E. M. Slart R. Mourits M. de Bock G. H. (2017a). Bone mineral density and fractures after surgical menopause: systematic review and meta-analysis. BJOG Int. J. Obstetrics and Gynaecol. 124, 1525–1535. 10.1111/1471-0528.14703 28436196

[B26] Fakkert I. E. van der Veer E. Abma E. M. Ledfrandt J. D. Wolffenbuttel H. R. Oosterwijk J. C. (2017b). Elevated bone turnover markers after risk-reducing salpingo-oophorectomy in women at increased risk for breast and ovarian cancer. PLoS One 12, e0169673. 10.1371/journal.pone.0169673 28060958 PMC5218401

[B27] Finkelstein J. S. Brockwell S. E. Mehta V. Greendale G. A. Sowers M. R. Ettinger B. (2008). Bone mineral density changes during the menopause transition in a multiethnic cohort of women. J. Clin. Endocrinol. and Metabolism 93, 861–868. 10.1210/jc.2007-1876 18160467 PMC2266953

[B28] Florencio-Silva R. Sasso G. R. D. S. Sasso-Cerri E. Simões M. J. Cerri P. S. (2015). Biology of bone tissue: structure, function, and factors that influence bone cells. BioMed Res. Int. 2015, 421746. 10.1155/2015/.421746 26247020 PMC4515490

[B29] Florencio-Silva R. Sasso G. R. S. Sasso-Cerri E. Simoes M. J. Cerri P. S. (2018). Effects of estrogen status in osteocyte autophagy and its relation to osteocyte viability in alveolar process of ovariectomized rats. Biomed. and Pharmacother. 98, 406–415. 10.1016/j.biopha.2017.12.089 29276969

[B30] Fujiwara Y. Piemontese M. Liu Y. Thostenson J. D. Xiong J. O’Brien C. A. (2016). RANKL (receptor activator of NFκB ligand) produced by osteocytes is required for the increase in B cells and bone loss caused by estrogen deficiency in mice. J. Biol. Chem. 291, 24838–24850. 10.1074/jbc.M116.742452 27733688 PMC5122756

[B31] Gallagher J. C. (2007). Effect of early menopause on bone mineral density and fractures. Menopause 14, 567–571. 10.1097/gme.0b013e31804c793d 17476146

[B32] Ginsburg E. S. Gao X. Shea B. F. Barbieri R. L. (1998). Half-life of estradiol in postmenopausal women. Gynecol. Obstetric Investigation 45, 45–48. 10.1159/000009923 9473164

[B33] Graham J. M. Ayati B. P. Holstein S. A. Martin J. A. (2013). The role of osteocytes in targeted bone remodeling: a mathematical model. PloS One 8, e63884. 10.1371/journal.pone.0063884 23717504 PMC3661588

[B34] Hadjidakis D. J. Kokkinakis E. P. Sfakianakis M. E. Raptis S. A. (2003). Bone density patterns after normal and premature menopause. Maturitas 44, 279–286. 10.1016/s0378-5122(03)00040-9 12697368

[B35] Harvey N. Dennison E. Cooper C. (2010). Osteoporosis: impact on health and economics. Nat. Rev. Rheumatol. 6, 99–105. 10.1038/nrrheum.2009.260 20125177

[B36] Hibler E. A. Kauderer J. Greene M. H. Rodriguez G. C. Alberts D. S. (2016). Bone loss after oophorectomy among high-risk women: an NRG oncology/gynecologic oncology group study. Menopause 23, 1228–1232. 10.1097/GME.0000000000000692 27433858 PMC5079806

[B37] Hofbauer L. Kuhne C. Viereck V. (2004). The OPG/RANKL/RANK system in metabolic bone diseases. J. Musculoskelet. Neuronal Interact. 4, 268–275. 15615494

[B38] Hsu S.-H. Chen L.-R. Chen K.-H. (2024). Primary osteoporosis induced by androgen and estrogen deficiency: the molecular and cellular perspective on pathophysiological mechanisms and treatments. Int. J. Mol. Sci. 25, 12139. 10.3390/ijms252212139 39596206 PMC11593909

[B39] Huber C. Collishaw S. Mosley J. R. Reeve J. Noble B. S. (2007). Selective estrogen receptor modulator inhibits osteocyte apoptosis during abrupt estrogen withdrawal: implications for bone quality maintenance. Calcif. Tissue Int. 81, 139–144. 10.1007/s00223-007-9049-6 17638036

[B40] Javed S. Sohail A. Nutini A. (2018). Integrative modeling of drug therapy and the bone turnover. Clin. Biomech. 60, 141–148. 10.1016/j.clinbiomech.2018.10.019 30359867

[B41] Jilka R. L. Takahashi K. Munshi M. Williams D. C. Roberson P. K. Manolagas S. C. (1998). Loss of estrogen upregulates osteoblastogenesis in the murine bone marrow: evidence for autonomy from factors released during bone resorption. J. Clin. Investigation 101, 1942–1950. 10.1172/JCI1039 9576759 PMC508781

[B42] Jörg D. J. Fuertinger D. H. Cherif A. Bushinsky D. A. Mermelstein A. Raimann J. G. (2022). Modeling osteoporosis to design and optimize pharmacological therapies comprising multiple drug types. eLife 11, e76228. 10.7554/elife.76228 35942681 PMC9363122

[B43] Kameda T. Mano H. Yuasa T. Mori Y. Miyazawa K. Shiokawa M. (1997). Estrogen inhibits bone resorption by directly inducing apoptosis of the bone-resorbing osteoclasts. J. Exp. Med. 186, 489–495. 10.1084/jem.186.4.489 9254647 PMC2199029

[B44] Karlafti E. Lampropoulou-Adamidou K. Tournis S. Trovas G. Triantafyllopoulos I. K. (2019). Effect of estrogen on bone cells: what is new? J. Res. Pract. Musculoskelet. Syst. 3, 113–122. 10.22540/JRPMS-03-113

[B45] Karlamangla A. S. Shieh A. Greendale G. A. (2021). “Chapter 15: hormones and bone loss across the menopause transition,” Editor Litwack G. (Cambridge, MA: Academic Press), 115, 401–417. 10.1016/bs.vh.2020.12.016 33706956

[B46] Kenkre J. S. Bassett J. H. D. (2018). The bone remodelling cycle. Ann. Clin. Biochem. 55, 308–327. 10.1177/0004563218759371 29368538

[B47] Khosla S. (2013). Pathogenesis of age-related bone loss in humans. J. Gerontol. Ser. A Biol. Sci. Med. Sci. 68, 1226–1235. 10.1093/gerona/gls163 22923429 PMC3826857

[B48] Khosla S. Hofbauer L. C. (2017). Osteoporosis treatment: recent developments and ongoing challenges. Lancet Diabetes and Endocrinol. 5, 898–907. 10.1016/S2213-8587(17)30188-2 28689769 PMC5798872

[B49] Khosla S. Burr D. Cauley J. Dempster D. W. Ebeling P. R. Felsenberg D. (2007). Bisphosphonate-associated osteonecrosis of the jaw: report of a task force of the American Society for Bone and Mineral Research. J. Bone Mineral Res. 22, 1479–1491. 10.1359/jbmr.0707onj 17663640

[B50] Khosla S. Oursler M. J. Monroe D. G. (2012). Estrogen and the skeleton. Trends Endocrinol. and Metabolism 23, 576–581. 10.1016/j.tem.2012.03.008 22595550 PMC3424385

[B51] Kim J. Han W. Park T. Kim E. J. Bang I. Lee H. S. (2020). Sclerostin inhibits Wnt signaling through tandem interaction with two LRP6 ectodomains. Nat. Commun. 11, 5357. 10.1038/s41467-020-19155-4 33097721 PMC7585440

[B52] Komarova S. V. Smith R. J. Dixon S. J. Sims S. M. Wahl L. M. (2003). Mathematical model predicts a critical role for osteoclast autocrine regulation in the control of bone remodeling. Bone 33, 206–215. 10.1016/s8756-3282(03).00157-1 14499354

[B53] Krause C. Korchynskyi O. de Rooij K. Weidauer S. E. de Gorter D. J. J. van Bezooijen R. L. (2010). Distinct modes of inhibition by sclerostin on bone morphogenetic protein and Wnt signaling pathways. J. Biol. Chem. 285, 41614–41626. 10.1074/jbc.M110.153890 20952383 PMC3009889

[B54] Larcher I. Scheiner S. (2021). Parameter reduction, sensitivity studies, and correlation analyses applied to a mechanobiologically regulated bone cell population model of the bone metabolism. Comput. Biol. Med. 136, 104717. 10.1016/j.compbiomed.2021.104717 34426166

[B55] Lemaire V. Cox D. R. (2019). Dynamics of bone cell interactions and differential responses to PTH and antibody-based therapies. Bull. Math. Biol. 81, 3575–3622. 10.1007/s11538-018-0533-0 30460589

[B56] Lemaire V. Tobin F. L. Greller L. D. Cho C. R. Suva L. J. (2004). Modeling the interactions between osteoblast and osteoclast activities in bone remodeling. J. Theor. Biol. 229, 293–309. 10.1016/j.jtbi.2004.03.023 15234198

[B57] Lerebours C. Buenzli P. Scheiner S. Pivonka P. (2016). A multiscale mechanobiological model of bone remodelling predicts site-specific bone loss in the femur during osteoporosis and mechanical disuse. Biomechanics Model. Mechanobiol. 15, 43–67. 10.1007/s10237-015-0705-x 26239380

[B58] Levenberg K. (1944). A method for the solution of certain non-linear problems in least squares. Q. Appl. Math. 2, 164–168. 10.1090/qam/10666

[B59] Li X. Zhang Y. Kang H. Liu W. Liu P. Zhang J. (2005). Sclerostin binds to LRP5/6 and antagonizes canonical Wnt signaling. J. Biol. Chem. 280, 19883–19887. 10.1074/jbc.M413274200 15778503

[B60] Li X. Ominsky M. S. Warmington K. S. Morony S. Gong J. Cao J. (2009). Sclerostin antibody treatment increases bone formation, bone mass, and bone strength in a rat model of postmenopausal osteoporosis. J. Bone Mineral Res. 24, 578–588. 10.1359/jbmr.081206 19049336

[B61] Looker A. C. Wahner H. W. Dunn W. L. Calvo M. S. Harris T. B. Heyse S. P. (1998). Updated data on proximal femur bone mineral levels of US adults. Osteoporos. Int. 8, 468–490. 10.1007/s001980050093 9850356

[B62] Marquardt D. W. (1963). An algorithm for least-squares estimation of nonlinear parameters. J. Soc. Industrial Appl. Math. 11, 431–441. 10.1137/0111030

[B63] Martin R. B. Burr D. B. Sharkey N. A. (1998). Skeletal tissue mechanics, New York, NY: Springer.

[B64] Martin M. Sansalone V. Cooper D. M. L. Forwood M. R. Pivonka P. (2019). Mechanobiological osteocyte feedback drives mechanostat regulation of bone in a multiscale computational model. Biomechanics Model. Mechanobiol. 18, 1475–1496. 10.1007/s10237-019-01158-w 31087221

[B65] Martínez-Reina J. Calvo-Gallego J. L. Pivonka P. (2021). Combined eeffects of exercise and denosumab treatment on local failure in post-menopausal osteoporosis–insights from bone remodelling simulations accounting for mineralisation and damage. Front. Bioeng. Biotechnol. 9, 635056. 10.3389/fbioe.2021.635056 34150724 PMC8212042

[B66] Matsuno K. Ueda K. Saito M. Kamii M. Tsuda A. Kawabata A. (2025). Pilot study of the effect of surgical menopause on bone mineral density and quality in patients with gynecological malignancies. J. Obstetrics Gynaecol. Res. 51, e16141. 10.1111/jog.16141 39530312 PMC11635186

[B67] McClung M. R. (2017). Sclerostin antibodies in osteoporosis: latest evidence and therapeutic potential. Ther. Adv. Musculoskelet. Dis. 9, 263–270. 10.1177/1759720X17726744 28974988 PMC5613857

[B68] McClung M. R. Grauer A. Boonen S. Bolognese M. A. Brown J. P. Diez-Perez A. (2014). Romosozumab in postmenopausal women with low bone mineral density. N. Engl. J. Med. 370, 410–420. 10.1056/.NEJMoa1305224 24382002

[B69] McNamara L. M. (2021). Osteocytes and estrogen deficiency. Curr. Osteoporos. Rep. 19, 592–603. 10.1007/.s11914-021-00702-x 34826091

[B70] Milovanovic P. Busse B. (2020). Phenomenon of osteocyte lacunar mineralization: indicator of former osteocyte death and a novel marker of impaired bone quality? Endocr. Connect. 9, R70–R80. 10.1530/EC-19-0531 32168472 PMC7159263

[B71] Mödder U. I. Clowes J. A. Hoey K. Peterson J. M. McCready L. Oursler M. J. (2010). Regulation of circulating sclerostin levels by sex steroids in women and in men. J. Bone Mineral Res. 26, 27–34. 10.1002/jbmr.128 20499362 PMC3132405

[B72] Moilanen A. Kopra J. Kröger H. Sund R. Rikkonen T. Sirola J. (2020). Characteristics of long-term femoral neck bone loss in postmenopausal women: a 25-year follow-up. J. Bone Mineral Res. 37, 173–178. 10.1002/jbmr.4444 34668233 PMC9298425

[B73] Nakashima T. Hayashi M. Fukunaga T. Kurata K. Oh-Hora M. Feng J. Q. (2011). Evidence for osteocyte regulation of bone homeostasis through RANKL expression. Nat. Med. 17, 1231–1234. 10.1038/nm.2452 21909105

[B74] Naqvi S. M. Panadero Pérez J. A. Kumar V. Verbruggen A. S. McNamara L. M. (2020). A novel 3D osteoblast and osteocyte model revealing changes in mineralization and pro-osteoclastogenic paracrine signaling during estrogen deficiency. Front. Bioeng. Biotechnol. 8, 601. 10.3389/fbioe.2020.00601 32656194 PMC7326002

[B75] Nelson A. C. Yeo E. F. Zhang Y. Cook C. V. Fischer-Holzhausen S. Keeler Bruce L. (2025a). Mathematical modeling of bone remodeling after surgical menopause. bioRxiv Preprint, 2025.10.19.683313. 10.1101/2025.10.19.683313 41278646 PMC12633214

[B76] Nelson A. C. Yeo E. F. Zhang Y. Cook C. V. Fischer-Holzhausen S. Keeler Bruce L. (2025b). SurgicalMenopauseBone. 10.5281/zenodo.17393743

[B77] Ohta H. Makita K. Komukai S. Nozawa S. (2002). Bone resorption *versus* estrogen loss following oophorectomy and menopause. Maturitas 43, 27–33. 10.1016/s0378-5122(02)00180-9 12270579

[B78] Ominsky M. S. Boyce R. W. Li X. Ke H. Z. (2017). Effects of sclerostin antibodies in animal models of osteoporosis. Bone 96, 63–75. 10.1016/j.bone.2016.10.019 27789417

[B79] Pansini F. Bagni B. Bonaccorsi G. Albertazzi P. Zanotti L. Farina A. (1995). Oophorectomy and spine bone density: evidence of a higher rate of bone loss in surgical compared with spontaneous menopause. Menopause 2, 109–116. 10.1097/00042192-199502020-00008

[B80] Parfitt A. (1976). The actions of parathyroid hormone on bone: relation to bone remodeling and turnover, calcium homeostasis, and metabolic bone diseases. Part I of IV parts: mechanisms of calcium transfer between blood and bone and their cellular basis: morphological and kinetic approaches to bone turnover. Metabolism 25, 809–844. 10.1016/0026-0495(76).90151-7 781470

[B81] Peris P. Alvarez L. Monegal A. Guañabens N. Durán M. Pons F. (1999). Biochemical markers of bone turnover after surgical menopause and hormone replacement therapy. Bone 25, 349–353. 10.1016/S8756-3282(99)00175-1 10495139

[B82] Pivonka P. Komarova S. V. (2010). Mathematical modeling in bone biology: from intracellular signaling to tissue mechanics. Bone 47, 181–189. 10.1016/j.bone.2010.04.601 20417739

[B83] Pivonka P. Zimak J. Smith D. W. Gardiner B. S. Dunstan C. R. Sims N. A. (2008). Model structure and control of bone remodeling: a theoretical study. Bone 43, 249–263. 10.1016/j.bone.2008.03.025 18514606

[B84] Pivonka P. Zimak J. Smith D. W. Gardiner B. S. Dunstan C. R. Sims N. A. (2010). Theoretical investigation of the role of the RANK–RANKL–OPG system in bone remodeling. J. Theor. Biol. 262, 306–316. 10.1016/j.jtbi.2009.09.021 19782692

[B85] Plotkin L. I. Bellido T. (2016). Osteocytic signalling pathways as therapeutic targets for bone fragility. Nat. Rev. Endocrinol. 12, 593–605. 10.1038/nrendo.2016.71 27230951 PMC6124897

[B86] Post T. M. Schmidt S. Peletier L. A. de Greef R. Kerbusch T. Danhof M. (2013). Application of a mechanism-based disease systems model for osteoporosis to clinical data. J. Pharmacokinet. Pharmacodynamics 40, 143–156. 10.1007/s10928-012-9294-9 23315144

[B87] Rachner T. D. Khosla S. Hofbauer L. C. (2011). Osteoporosis: now and the future. Lancet 377, 1276–1287. 10.1016/S0140-6736(10)62349-5 21450337 PMC3555696

[B88] Rattanakul C. Lenbury Y. Krishnamara N. Wollkind D. J. (2003). Modeling of bone formation and resorption mediated by parathyroid hormone: response to estrogen/PTH therapy. Biosystems 70, 55–72. 10.1016/s0303-2647(03).00040-6 12753937

[B89] Recker R. R. Benson C. T. Matsumoto T. Bolognese M. A. Robins D. A. Alam J. (2015). A randomized, double-blind phase 2 clinical trial of blosozumab, a sclerostin antibody, in postmenopausal women with low bone mineral density. J. Bone Mineral Res. 30, 216–224. 10.1002/jbmr.2351 25196993

[B90] Robling A. G. Castillo A. B. Turner C. H. (2006). Biomechanical and molecular regulation of bone remodeling. Annu. Rev. Biomed. Eng. 8, 455–498. 10.1146/annurev.bioeng.8.061505.095721 16834564

[B91] Robling A. G. Niziolek P. J. Baldridge L. A. Condon K. W. Allen M. R. Alam I. (2008). Mechanical stimulation of bone *in vivo* reduces osteocyte expression of Sost/sclerostin. J. Biol. Chem. 283, 5866–5875. 10.1074/jbc.M705092200 18089564

[B92] Rodan G. A. Fleisch H. A. (1996). Bisphosphonates: mechanisms of action. J. Clin. Investigation 97, 2692–2696. 10.1172/JCI118722 8675678 PMC507360

[B93] Rodriguez M. Shoupe D. (2015). Surgical menopause. Endocrinol. Metabolism Clin. N. Am. 44, 531–542. 10.1016/j.ecl.2015.05.003 26316241

[B94] Rogers M. J. Frith J. C. Luckman S. P. Coxon F. P. Benford H. L. Mönkkönen J. (1999). Molecular mechanisms of action of bisphosphonates. Bone 24, 73S–79S. 10.1016/s8756-3282(99)00070-8 10321934

[B95] Ruiz-Lozano R. Calvo-Gallego J. L. Pivonka P. McDonald M. M. Martínez-Reina J. (2024). An *in silico* approach to elucidate the pathways leading to primary osteoporosis: age-related vs. postmenopausal. Biomechanics Model. Mechanobiol. 23, 1393–1409. 10.1007/s10237-024-01846-2 38700787 PMC11584493

[B96] Russell R. G. Croucher P. I. Rogers M. J. (1999). Bisphosphonates: pharmacology, mechanisms of action and clinical uses. Osteoporos. Int. 9. 10.1007/pl00004164 10525729

[B97] Santos A. Bakker A. D. Klein-Nulend J. (2009). The role of osteocytes in bone mechanotransduction. Osteoporos. Int. 20, 1027–1031. 10.1007/s00198-009-0858-5 19340507

[B98] Sarafrazi N. Wambogo E. A. Shepherd J. A. (2021). Osteoporosis or low bone mass in older adults: united States, 2017–2018. NCHS Data Briefs 405. 1–7. 10.15620/cdc:103477 34029181

[B99] Scheiner S. Pivonka P. Hellmich C. (2013). Coupling systems biology with multiscale mechanics, for computer simulations of bone remodeling. Comput. Methods Appl. Mech. Eng. 254, 181–196. 10.1016/j.cma.2012.10.015

[B100] Scheiner S. Pivonka P. Smith D. W. Dunstan C. R. Hellmich C. (2014). Mathematical modeling of postmenopausal osteoporosis and its treatment by the anti-catabolic drug denosumab. Int. J. Numer. Methods Biomed. Eng. 30, 1–27. 10.1002/cnm.2584 24039120 PMC4291103

[B101] Schmidt S. Post T. M. Peletier L. A. Boroujerdi M. A. Danhof M. (2011). Coping with time scales in disease systems analysis: application to bone remodeling. J. Pharmacokinet. Pharmacodynamics 38, 873–900. 10.1007/s10928-011-9224-2 22028207 PMC3230316

[B102] Seeman E. (2013). Age-and menopause-related bone loss compromise cortical and trabecular microstructure. J. Gerontol. Ser. A Biomed. Sci. Med. Sci. 68, 1218–1225. 10.1093/gerona/glt071 23833200

[B103] Shane E. Burr D. Abrahamsen B. Adler R. A. Brown T. D. Cheung A. M. (2014). Atypical subtrochanteric and diaphyseal femoral fractures: second report of a task force of the American Society for Bone and Mineral Research. J. Bone Mineral Res. 29, 1–23. 10.1002/jbmr.1998 23712442

[B104] Shieh A. Ishii S. Greendale G. A. Cauley J. A. Lo J. C. Karlamangla A. S. (2016). Urinary N-telopeptide and rate of bone loss over the menopause transition and early postmenopause. J. Bone Mineral Res. 31, 2057–2064. 10.1002/jbmr.2889 27322414 PMC5407063

[B105] Sipos W. Pietschmann P. Rauner M. Kerschan-Schindl K. Patsch J. (2009). Pathophysiology of osteoporosis. Wien. Med. Wochenschr. 159, 230–234. 10.1007/s10354-009-0647-y 19484205

[B106] Suen P. K. Qin L. (2016). Sclerostin, an emerging therapeutic target for treating osteoporosis and osteoporotic fracture: a general review. J. Orthop. Transl. 4, 1–13. 10.1016/j.jot.2015.08.004 30035061 PMC5987014

[B107] The North American Menopause Society (2021). Management of osteoporosis in postmenopausal women: the 2021 position statement of the North American Menopause Society. Menopause 28, 973–997. 10.1097/GME.0000000000001831 34448749

[B108] Tian X. Jee W. S. Li X. Paszty C. Ke H. Z. (2011). Sclerostin antibody increases bone mass by stimulating bone formation and inhibiting bone resorption in a hindlimb-immobilization rat model. Bone 48, 197–201. 10.1016/j.bone.2010.09.009 20850580

[B109] Tomkinson A. Reeve J. Shaw R. W. Noble B. S. (1997). The death of osteocytes *via* apoptosis accompanies estrogen withdrawal in human bone. J. Clin. Endocrinol. and Metabolism 82, 3128–3135. 10.1210/jcem.82.9.4200 9284757

[B110] Tomkinson A. Gevers E. F. Wit J. M. Reeve J. Noble B. S. (1998). The role of estrogen in the control of rat osteocyte apoptosis. J. Bone Mineral Res. 13, 1243–1250. 10.1359/jbmr.1998.13.8.1243 9718192

[B111] Trichilo S. Scheiner S. Forwood M. Cooper D. M. L. Pivonka P. (2019). Computational model of the dual action of PTH – application to a rat model of osteoporosis. J. Theor. Biol. 473, 67–79. 10.1016/j.jtbi.2019.04.020 31009612

[B112] van Oers R. F. Ruimerman R. Tanck E. Hilbers P. A. J. Huiskes R. (2008). A unified theory for osteonal and hemi-osteonal remodeling. Bone 42, 250–259. 10.1016/j.bone.2007.10.009 18063436

[B113] Whitaker M. Guo J. Kehoe T. Benson G. (2012). Bisphosphonates for osteoporosis—where do we go from here? N. Engl. J. Med. 366, 2048–2051. 10.1056/NEJMp1202619 22571168

[B114] Wijenayaka A. R. Kogawa M. Lim H. P. Bonewald L. F. Findlay D. M. Atkins G. J. (2011). Sclerostin stimulates osteocyte support of osteoclast activity by a rankl-dependent pathway. PloS One 6, e25900. 10.1371/journal.pone.0025900 21991382 PMC3186800

[B115] Yasui T. Uemura H. Tomita J. Miyatani Y. Yamada M. Kuwahara A. (2007). Change in serum undercarboxylated osteocalcin concentration in bilaterally oophorectomized women. Maturitas 56, 288–296. 10.1016/j.maturitas.2006.09.002 17030103

[B116] Zhai G. Hart D. Valdes A. Kato B. Richards J. Hakim A. (2008). Natural history and risk factors for bone loss in postmenopausal caucasian women: a 15-year follow-up population-based study. Osteoporos. International 19, 1211–1217. 10.1007/s00198-008-0562-x 18305885

[B117] Zhang D. Hu M. Chu T. Lin L. Wang J. Li X. (2016). Sclerostin antibody prevented progressive bone loss in combined ovariectomized and concurrent functional disuse. Bone 87, 161–168. 10.1016/j.bone.2016.02.005 26868528 PMC4862887

